# The Influence of Feedback Content and Feedback Time on Multimedia Learning Achievement of College Students and Its Mechanism

**DOI:** 10.3389/fpsyg.2021.706821

**Published:** 2021-12-09

**Authors:** Sayipujiamali Taxipulati, Hai-Dong Lu

**Affiliations:** School of Psychology, Northeast Normal University, Changchun, China

**Keywords:** multimedia learning, feedback time, feedback content, cognitive load, eye movement

## Abstract

This study investigated the content of feedback (adaptive, elaborated, and knowledge of correct response, KCR) and time (Immediate and Delayed) influences on multimedia learning of college students. Students from the Northeast Normal University (*N* = 157) were randomly assigned to one of the six experimental conditions. We tried to explain the influence mechanisms of different feedback effects through subjectively reported motivation, cognitive load, and eye movement trajectory during the feedback period. The results showed that (1) different feedback methods in terms of feedback time and feedback content have significantly different effects on scores. Among them, scores of the immediate feedback group were significantly higher than those of the delayed feedback group, and the scores of the adaptive feedback (AF) group were significantly higher than those of the elaborated feedback (EF) group and the knowledge of the correct response feedback group. (2) Different types of feedback contents have significantly different effects on motivation. The motivation scores reported by the AF group and EF group were significantly higher than those reported by the knowledge of the correct response feedback group. (3) Different feedback methods in terms of feedback time and feedback content had significantly different effects on subjective germane cognitive load reports. Among them, the germane cognitive load score of the immediate feedback group was significantly higher than that of the delayed feedback group. The germane cognitive load scores reported by the AF group were significantly higher than those reported by the EF group and knowledge of the correct response feedback group. (4) The germane cognitive load plays a partial mediating role between the AF and post-test scores. (5) Different feedback methods in feedback time have different effects on eye movement fixation trajectory, which shows that the subjects in the immediate feedback group were significantly less than those in the delayed feedback group in fixation count and fixation time in the interest area of the stem. (6) Consistent with our hypothesis, different feedback methods in feedback content have different effects on eye movement trajectory. In summary, the results show that the AF initiated in this study has a positive effect on multimedia learning of college students; it not only provides empirical evidence for cognitive load theory but also helps educators design adaptive learning feedback according to responses of students.

## Introduction

With the development of computer and network technology, computers have gradually become an important tool for learning and teaching. The word multimedia learning is becoming a hot topic in the field of education. Compared with traditional classroom learning, multimedia learning has the powerful function of providing information about the current state of learning and how to eliminate the discrepancy between the current state and the goal, thus allowing learners to adjust their cognition or behavior to promote learning (Mory, 2004; Hattie and Timperley, 2007). However, it is difficult to provide personalized automatic feedback to each student in traditional classroom teaching; therefore, the role of feedback in multimedia learning has been widely studied by researchers in the field of education. Although such multimedia environments have been used for a long time, the effects of automated feedback have been widely studied, and research evidence is inconsistent in many respects (Kluger and DeNisi, 1996; Van der Kleij et al., 2015).

In addition, there are two main problems in previous research on multimedia learning feedback. First, most previous studies have compared the effects of elaborated feedback (EF) and simple feedback with different contents on learning, but few studies have considered providing adaptive feedback (AF) to learners according to the characteristics of learners, the purpose of feedback, and the characteristics of feedback tasks. Empirical research on this aspect lags far behind the application of multimedia learning feedback in real life. At present, many learning applications in the market have developed the function of providing AF according to learning progress or answering questions of the learners. Narciss et al. (2014) pointed out that due to the differences in personal characteristics (such as prior knowledge, learning progress, and learning preferences) and other external variables of learners, providing AF to learners in a multimedia learning environment is a promising method (Narciss et al., 2014). Attali and Van der Kleij (2017) used isomorphic math problems nested in practice and tested to study the influence of different feedbacks received in practice on task performance in the test stage and divided them into groups according to the right and wrong answers and feedback types in the practice stage and compared the influence of different feedback received after correct and incorrect answers on problem-solving performance in the test stage. The results showed that after the subjects answered the wrong questions in the practice stage, those who received EF (correct answer + analysis) performed better in the test stage than those who received the knowledge of correct response (KCR) feedback. However, when they answered the questions correctly in the practice stage, there was no significant difference in the performance of the two groups of subjects who received the EF and KCR feedback in the test stage (Attali, 2015; Attali et al., 2016; Attali and Van der Kleij, 2017).

The characteristics of certain feedback largely determine its effectiveness, and research on this topic has suggested that feedback content is the most important factor in learning (Bangert-Drowns et al., 1991; Shute, 2008). Shute (2008) distinguished different forms of feedback content based on their complexity. The three most frequently studied feedback types in the literature are as follows.

(1) Knowledge of results (KR): verifying the correctness of the response (such as your answer is right/wrong);
(2) Knowledge of correct response: providing the correct response (e.g., the correct answer is A/B and so on);
(3) Elaborated feedback: providing additional information, such as strategic hints, an explanation, or a worked-out example (such as your answer is right/wrong, because…).

In general, KR and KCR are called simple feedback (Bennett, 2015). According to the cognitive load theory, when learning materials are organized or presented in an inappropriate way or redundant information is included in the learning materials. Cognitive resources are used to engage in activities unrelated to learning, that is, to improve external cognitive load. Three types of cognitive load have been identified in previous studies: “intrinsic cognitive load referred to the complexity of the information being processed and was related to the concept of element interactivity” (Sweller et al., 2019). Element interactivity refers to the number of elements that learners must process simultaneously (Sweller, 2010) in their working memory, which mainly depends on the combination of the complexity of the learning material and the level of knowledge of the learner. Extraneous cognitive load refers to the unnecessary load that interferes with learning, which mainly results from improper instructional design (Sweller et al., 2019). Germane cognitive load was defined as the working memory resources related to schema acquisition and automation, which is the load that promotes learning (Paas et al., 2003).

Based on cognitive load theory and the results of Attali and Van der Kleij (2017), we put forward the concept of AF in this study, that is, to provide KCR feedback when the subjects answer correctly and provide EF when the subjects answer incorrectly. Previous research on AF has also found the importance of such feedback based on learner needs. Lin et al. (2021) developed a badminton teaching system using wearable technology to improve badminton teaching and learning. This system can provide similarity scores automatically by comparing movement and strength of a student while playing badminton with a well-trained expert model. The results demonstrate the importance of providing instant and AF in motor skill learning (Lin et al., 2021).

Second, most of the assumptions about the feedback mechanism were based on theoretical discussions, which needed the support of systematic empirical research. The meaning of the mechanism here is to interpret the relationship or links or association between variables, such as the role of emotion in the impact of feedback on reading achievement (Lipnevich et al., 2021). Explain the locus of the effect, such as why errors (Potts et al., 2019) or speculations (Zawadzka and Hanczakowski, 2019), can be beneficial in the learning process. At present, some studies explain the mechanism of feedback by measuring cognitive load, learning motivation (Jurik et al., 2014), and other variables of students. For example, Abbas and North (2018) examined the effects of feedback (KR) after good and poor performance on self-efficacy and intrinsic motivation when learning easy and more difficult motor tasks. The KR-good group showed the highest levels of self-efficacy and intrinsic motivation, relative to the other two feedback groups, and more accurate putting performance (Abbas and North, 2018). Another study found that students who received problem-solving question prompt and corrective feedback achieved better performance and perceived less cognitive load (Huang et al., 2015). A study that investigated the effects of the presence of an animated agent and different types of feedback on learning, motivation, and cognitive load in a multimedia learning environment designed to teach science content found that participants who learned with the animated agent that delivered EF had significantly higher scores on a learning measure compared to participants who learned with an agent that provided simple feedback (KR) (Lin et al., 2013). Another study that investigated the complexity of EF (additional instructional information of the correct answer) and item format as influences on learning in a computer-based formative assessment showed that detailed explanations resulted in lower extraneous cognitive load but higher germane cognitive load and learning motivation than cues; constructed-response items resulted in lower intrinsic and extraneous cognitive load but higher germane cognitive load than multiple-choice items. Furthermore, feedback complexity has an indirect effect on transfer performance via the germane cognitive load (Fyfe et al., 2012; Golke et al., 2015; Fyfe and Rittle-Johnson, 2016; Finn et al., 2018; Wang et al., 2019).

However, the measurement of these variables is limited to subjective reports. Studies have shown that changes in eye movement are closely related to cognitive load in the process of learning and problem solving (Moreno, 2004; Kornell and Rhodes, 2013; Zu et al., 2019). For example, Zu et al. (2019) found significant correlations between the manipulation of extraneous load and shorter mean fixation durations, longer mean saccade lengths, and higher blink rates. Thus, the mean fixation duration, mean saccade length, and blink rate were sensitive to extraneous load. This study also shows significant relationships between both the extraneous load manipulation and the proxy measure of germane load, with the ratio of pupil size change, but in opposite directions. Specifically, students in the extraneous load condition showed a greater ratio of pupil size change, while students evidencing greater germane load showed a smaller ratio of pupil size change. Further analysis of different areas of interest (AOIs) showed that students with higher germane load showed significantly higher dwell times compared to those with lower germane load (Zu et al., 2019). According to the results of previous studies, we put the content of the feedback stage appear on the screen is divided into two interest areas, respectively, is problem stem interest area and feedback interest area, select fixation time of subjects, fixation count of the two interest areas, the regression count between the two interest areas, and pupil size as the objective index of cognitive processing and the distribution of attention of subjects. Fixation time refers to the amount of time spent by the gaze on the stimulus (usually 200–300 ms). Fixation count of the interest area is the number of times that the fixation point falls into a certain area of interest within a certain time. The regression count is the number of looking back between different AOIs reflects the process of connection and integration between the information of two AOIs. Pupil size refers to the change in pupil size in response to gaze stimuli (Ozeri-Rotstain et al., 2020).

In addition to the feedback content, the timing of feedback is a widely studied but poorly understood variable in the feedback process. In most studies in the feedback literature, a distinction is made between immediate and delayed feedback (Shute, 2008; Fyfe, 2016). Van der Kleij et al. (2012) proposed a univocal definition of feedback timing in computer-based assessments: immediate means directly after the learner responded to the item and delayed means directly after responding to all the items in a test. This definition was used in this study.

In summary, this study builds on currently available evidence regarding the effectiveness of feedback in multimedia learning environments. Based on cognitive load theory, we examined the effects of feedback content and feedback timing on learning outcomes by investigating the mediating roles of cognitive load and motivation of learners. Distinct from previous research in the field, we aimed to construct a more comprehensive understanding of the effects of providing AF based on learner responses due to cognition and motivation. We expect the results of this study to provide valuable empirical evidence on how to design AF for learners in the context of multimedia learning.

### Aims of This Study

Under the framework of cognitive load theory, this study aimed to explore the influence of different feedback contents and feedback times on problem-solving performance of learners in the testing stage and further explore the mechanism of cognitive load and motivation in the influence of feedback on performance. The specific purposes of this study are as follows:

First, do different types of feedback designed according to feedback content and feedback time have significantly different effects on problem-solving performance of learners in the test stage?

Second, do different types of feedback designed according to feedback content and feedback time have significantly different effects on subjective cognitive load and objective eye-tracking trajectory of learners?

Third, do different types of feedback designed according to feedback content and feedback time have different effects on motivation of learners?

Fourth is the influence of different types of feedbacks on problem-solving performance of learners in the test stage realized through the mediation of cognitive load and motivation?

### Research Hypotheses

H1: Different types of feedback designed according to feedback content and feedback time have significantly different effects on problem-solving performance of learners in the test stage.
H1a: The performance of solving problems in the AF group is higher than those in the EF group and the knowledge of the correct response feedback group.
H1b: The problem-solving performance of the immediate feedback group is higher than that of the delayed feedback group.
H2: Different types of feedback have significantly different effects on subjective cognitive load and objective eye-tracking trajectories of learners.
H2a: The external cognitive load reported by the AF group and the EF group is lower than that of the knowledge of the correct response feedback group; if attention of learners to the interest area of the stem is regarded as an objective indicator of external cognitive load, then the fixation time and fixation count of the subjects in the AF group and the EF group are also lower than those in the knowledge of the correct response feedback group.
H2b: The germane cognitive load reported by the AF group and EF group was higher than that of the knowledge of the correct response feedback group. If attention of learners to the feedback interest area and the regression between the problem stem interest area and the feedback interest area are taken as objective indicators of the germane cognitive load, then the fixation time and fixation count of the AF group and the EF group and the regression between the problem stem interest area and the feedback interest area are also higher than those of the knowledge of the correct response feedback group.
H2c: Compared with the delayed feedback group, the immediate feedback group reported lower external cognitive load and higher germane cognitive load. Accordingly, compared with the subjects in the delayed feedback group, the subjects in the immediate feedback group pay less attention to the problem stem interest area, but pay more attention to the feedback interest area, and look back more times between the feedback interest area and the problem stem interest area.
H3: Different types of feedbacks have significantly different effects on the motivation of learners.
H3a: The learning motivation of the AF group was higher than that of the EF group and the KCR feedback group.
H3b: The learning motivation of the immediate feedback group is higher than that of the delayed feedback group.
H4: The influence of different types of feedback on problem-solving performance of learners in the test stage is realized through the mediation of cognitive load and motivation.

## Materials and Methods

### Participants

The research participants of this study were 168 undergraduate and graduate students from the Northeast Normal University. Finally, through data screening, 157 effective subjects (38 men and 119 women) aged between 17 and 26 years were included. All the participants were randomly assigned to six groups and performed the experiments in the experimental group, and each group of the specific distribution was shown in Table 1.

**TABLE 1:** Summary of design of experimental groups.

**Group**	**Feedback time**	**Feedback content**	***N***
1	Immediate	Adaptive feedback	26
2	Immediate	Elaborated feedback	26
3	Immediate	Knowledge of correct response feedback	26
4	Delayed	Adaptive feedback	26
5	Delayed	Elaborated feedback	27
6	Delayed	Knowledge of correct response feedback	26

*Immediate means directly after the learner responded to the item, and delayed means directly after responding to all the items in a test. This definition will be used in the present study.*

*Knowledge of results (KR): question stem + correct answer. Elaborated feedback (EF): question stem + correct answer + correct answer resolution. Adaptive feedback (AF): if the answer of the subject is correct, provide knowledge of correct response feedback; if the answer is wrong, provide elaborated feedback.*

### Material

The experimental materials are the graphic reasoning questions in the National Civil Service Examination vocational test of administrative ability, which tests the ability of the candidates to observe, abstract, and reason. According to the purpose of the experiment, part of the quantity rule was selected as the final experimental material.

First, the examinator randomly selected 60 questions from a civil service examination training app. Then, through the pre-test, 21 questions with a difficulty of 0.3–0.7 were selected as the final experimental materials, and the time needed to complete these questions, feedback presentation time, and other specific details were further determined. Among them, 3 questions were randomly selected for the pre-test, 10 questions for practice, and 8 questions for post-test materials. The order of the presentation of these questions was random. The details of the materials are as follows:

Pre-test materials: Three reasoning questions were randomly selected from the question bank, and the subjects were asked whether they had participated in the civil service exam or learned similar knowledge related to graphic reasoning before conducting the experiment.

Learning materials: Ten slides, i.e., two pages of the introduction of problem-solving skills and eight pages of sample questions. The sample questions were presented in the form of questions on the left and analysis on the right. To ensure a consistent learning time for each subject, the slides were set to play automatically; each page played for 30 s, and the total learning time was 5 min.

Exercise material: Ten reasoning questions related to the learning material.

Feedback content material: KCR feedback: question stem + correct answer;

Elaborated feedback: question stem + correct answer + correct answer resolution;

Adaptive feedback: if the answer of the subject is correct, provide KCR feedback; if the answer is wrong, provide EF;

Post-test material: Eight reasoning questions related to learning and exercise materials.

Situational learning motivation scale: Referred to the scale of learning motivation adapted from Keller’s (2010) by Wang et al. (2019). Participants rated 11 items on a 5-point scale ranging from 1 (*strongly disagree*) to 5 (*strongly agree*), with higher scores indicating a higher motivation level. Cronbach’s alpha for the combined subscales in the study of Wang et al. (2019) was 0.87, and in this study, it was 0.90, indicating that the reliability was better.

Cognitive load scale: The Chinese version of the cognitive load scale compiled by Leppink et al. (2013) was adopted. The original scale included three dimensions of intrinsic cognitive load (four items), external cognitive load (four items), and germane cognitive load (five items), with a total of 13 questions. According to the purpose of this experiment, this study selected nine questions of external cognitive load and germane cognitive load as a tool to measure the subjective reported cognitive load. The scale uses a 5-point scale, 1 (*strongly disagree*) to 5 (*strongly agree*), and the higher the score, the higher the cognitive load. In this study, the Cronbach’s coefficient of the scale was 0.78, indicating that its reliability was within an acceptable range.

Eye movement indicators: According to the results of previous studies, the content of the feedback stage that appears on the screen is divided into two interest areas: problem stem interest area and feedback interest area, select subjects’ fixation time, fixation count of the two interest areas, the regression count between the two interest areas, and pupil size as the objective index of cognitive processing and the distribution of attention of the subjects.

### Procedure

This study uses E-Builder to write the computer-based experiment program, such as the pre-test stage, learning stage, practice stage, and the post-test stage, and the whole experiment time is about 30 min. The main processes are as follows:

First, the experimental procedure was briefly and clearly explained to the subjects.

Second, after the subjects were randomly assigned to an experimental group according to the number, they completed the pre-test stage.

Third, based on the pre-test results, if they met our requirements, they entered the learning stage.

Then, the subject sat in front of the eye tracker, calibrated the right eye, and started to perform exercises once the calibration was correct. In the practice stage, participants in the immediate feedback group received feedback for each problem they completed, while those in the delayed feedback group they received feedback after they finished all the exercises.

At the end of the exercise period, the subjects left the front of the eye tracker and continued to complete the post-test on the computer. In the post-test stage, the cognitive load scale, situational motivation scale, and post-test questions were completed successively.

Finally, the subjects were paid accordingly, and according to their performance, they could choose a small gift (key chain, handkerchief paper, and signature pen) they liked and then left the lab.

### Experimental Design and Data Analyses

A 2 feedback times (immediate/delayed) × 3 feedback contents (KCR/EF/AF feedback) between-subject design was used in the study. The independent variables were feedback time and feedback content, while dependent variables were post-test scores, situational motivation, cognitive load (external cognitive load and germane cognitive load), fixation time, fixation count, regression count, and pupil size. The control variables were the difficulty of the questions and the prior knowledge level of the subjects.

Descriptive statistical analysis, correlation analysis, mediating effect analysis, and variance analysis were performed using SPSS 26.0.

## Results

### Descriptive Statistical Analysis

The descriptive statistics of six groups of subjects on each dependent variable was shown in Table 2.

**TABLE 2:** Descriptive statistical analysis.

**Group**	**IM-AF**		**IM-EF**		**IM-KCR**		**DE-AF**		**DE-EF**		**DE-KCR**	
**Dependent variable**	***M***	***SD***	***M***	***SD***	***M***	***SD***	***M***	***SD***	***M***	***SD***	***M***	***SD***
Post-test score	5.00	1.30	4.46	1.53	4.54	1.48	4.85	1.71	3.93	1.69	3.69	1.81
Situational learning motivation	3.36	0.64	3.19	0.78	3.06	0.76	3.39	0.56	3.23	0.69	2.80	0.77
External cognitive load	2.62	0.99	2.59	0.71	2.62	0.80	2.42	0.57	2.65	0.72	2.73	1.01
Germane cognitive load	4.00	0.41	3.88	0.60	3.81	0.56	3.92	0.48	3.81	0.55	3.48	0.75

### Post-test Results of Learners Under Different Feedback Conditions

**FIGURE 1:**
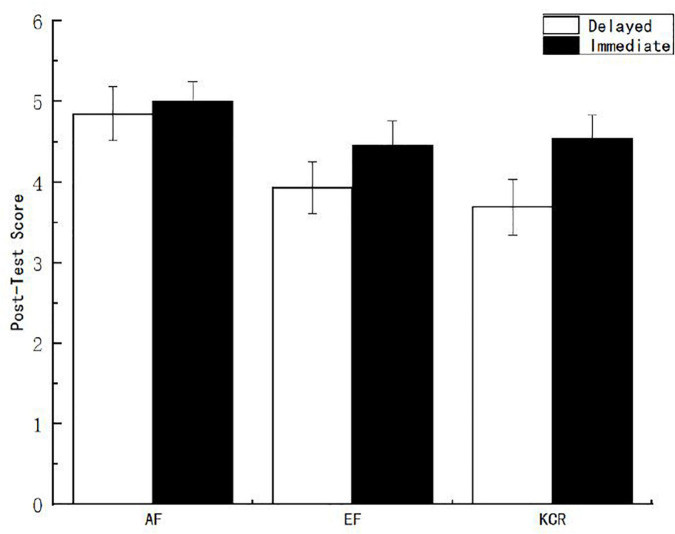
Post-test scores of learners under different feedback conditions.

To explore the influence of feedback content and feedback time on learning, a variance analysis was conducted on the post-test scores under different feedback conditions. The results showed that the main effect of feedback time on post-test scores of learners was significant [*F*_(1, 151)_ = 4.046, *p* < 0.05, η^2^*_*p*_* = 0.024]. According to Figure 1, the scores of the immediate feedback group are better than those of the delayed feedback group. The main effect of feedback content on post-test scores of learners was also significant [*F*_(2, 151)_ = 4.069, *p* < 0.05, η^2^*_*p*_* = 0.049]. After multiple comparisons of the feedback content, it was found that the post-test scores of the AF group were significantly higher than those of the EF group and the knowledge of the correct response feedback group.

**FIGURE 2:**
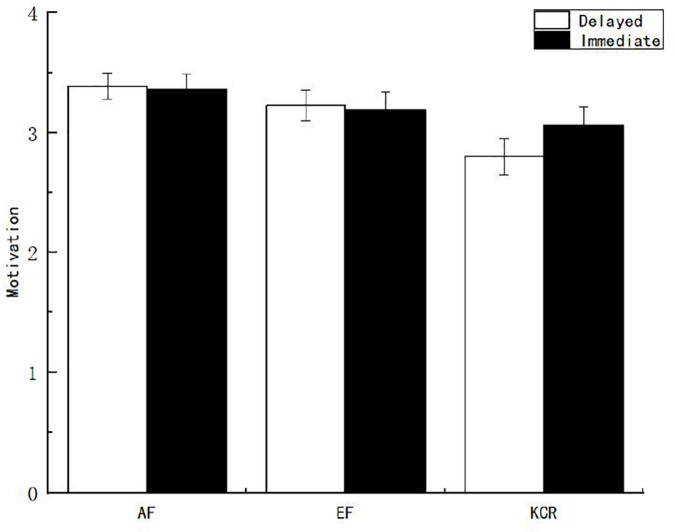
Situational learning motivation of learners under different feedback conditions.

To explore the influence of feedback content and feedback time on motivation, variance analysis was conducted on the scores of situational learning motivation reported by subjects under different feedback conditions, and it was found that the main effect of feedback time was not significant (*p* > 0.05). The main effect of feedback content was significant [*F*_(2, 151)_ = 5.268, *p* < 0.05, η^2^*_*p*_* = 0.061]. After further multiple comparisons of the feedback contents, it was found that the scores of situational learning motivation reported by the AF and EF groups were significantly higher than those reported by the knowledge of the correct response feedback group (see Figure 2).

### Cognitive Load Results Reported by Learners Under Different Feedback Conditions

To explore the influence of feedback content and time on cognitive load, variance analysis was conducted on external cognitive load and associated cognitive load under different feedback conditions, and it was found that there was no significant difference between the external cognitive load scores reported by subjects under different feedback times and contents.

**FIGURE 3:**
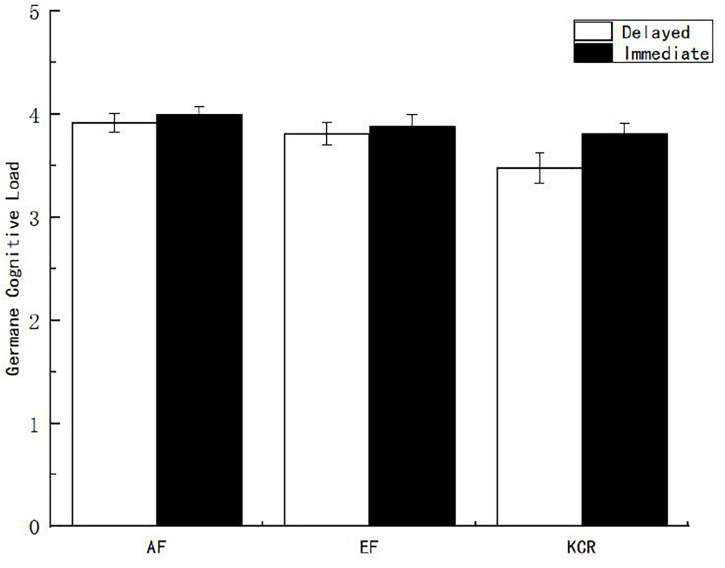
Germane cognitive load of learners under different feedback conditions.

The main effect of feedback time on the germane cognitive load was marginally significant [*F*_(1, 151)_ = 2.996, *p* = 0.085, η^2^*_*p*_* = 0.029], which indicates that the germane cognitive load of the immediate feedback group was higher than that of the delayed feedback group (see Figure 3). The main effect of feedback content on the related cognitive load was also significant [*F*_(2, 151)_ = 4.213, *p* < 0.05, η^2^*_*p*_* = 0.049]. After further multiple comparisons of feedback contents, it was found that the germane cognitive load scores reported by the AF group were significantly higher than those reported by the EF group and the knowledge of the correct response feedback group.

### Analysis of the Mechanism of Feedback Types

To further explore the mechanism of feedback at different times and content on performance of learners in the post-test stage, we took AF as 1 and the other two as 0; EF is 1 and the other two are 0; the KCR feedback is 1 and the other two are 0. In this way, we set virtual variables. The point two-column correlation analysis was carried out between different feedback times and content, post-test scores, external cognitive load, germane cognitive load, and motivation. The specific results are presented in Table 3.

**TABLE 3:** Correlation analysis.

**Variable**		**1**	**2**	**3**	**4**	**5**	**6**	**7**	**8**
1	Feedback time	1							
2	AF	0.00	1						
3	KCR	0.00	−0.50**	1					
4	EF	–0.00	−0.50**	−0.50**	1				
5	Post-test score	0.16*	0.22**	–0.13	–0.10	1			
6	Motivation	0.04	0.20*	−0.24**	0.04	0.08	1		
7	Germane cognitive load	0.13	0.18*	−0.21**	0.03	0.26**	0.60**	1	
8	External cognitive load	0.00	–0.07	0.06	0.01	–0.08	−0.58**	−0.40**	1

**Significant correlation at 0.01 level (bilateral).

*Significant correlation at the level of 0.05 (bilateral).

**FIGURE 4:**
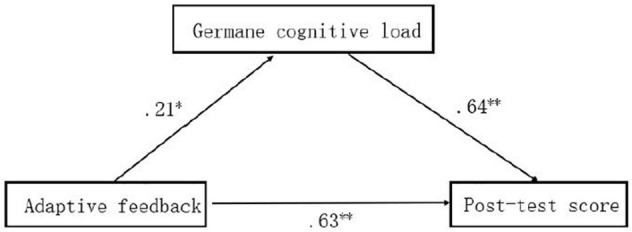
Mediating model of germane cognitive load. *Significant correlation at the level of 0.05 (bilateral). **Significant correlation at 0.01 level (bilateral).

According to the results of correlation analysis, we further test the mediating effect with the hierarchical regression method, in which the post-test result is a dependent variable, the AF is an independent variable, and the germane cognitive load is an intermediary variable. The results show that the germane cognitive load plays a partial mediating role in the influence of AF on the post-test result, as shown in Figure 4.

### Fixation Behavior of Learners on the Interest Area of the Stem Under Different Feedback Conditions

First, the subjects performed a descriptive statistical analysis of the eye movement indexes in the interest area of the stem, and the results are shown in Table 4.

**TABLE 4:** Descriptive statistical analysis.

	**IM-AF**		**IM-EF**		**IM-KCR**		**DE-AF**		**DE-EF**		**DE-KCR**	
**Dependent variable**	***M***	***SD***	***M***	***SD***	***M***	***SD***	***M***	***SD***	***M***	***SD***	***M***	***SD***
Pupil size	700.83	225.60	643.47	182.95	716.32	202.31	668.79	172.42	623.26	162.78	662.31	185.15
Fixation time (*ms*)	5471.39	1680.63	4645.80	1831.58	8490.29	3687.61	6840.07	2998.98	6282.47	2414.24	11076.71	3034.19
Fixation count	24.11	6.57	20.27	7.02	35.27	14.98	31.11	12.17	27.76	9.57	43.23	13.31

**FIGURE 5:**
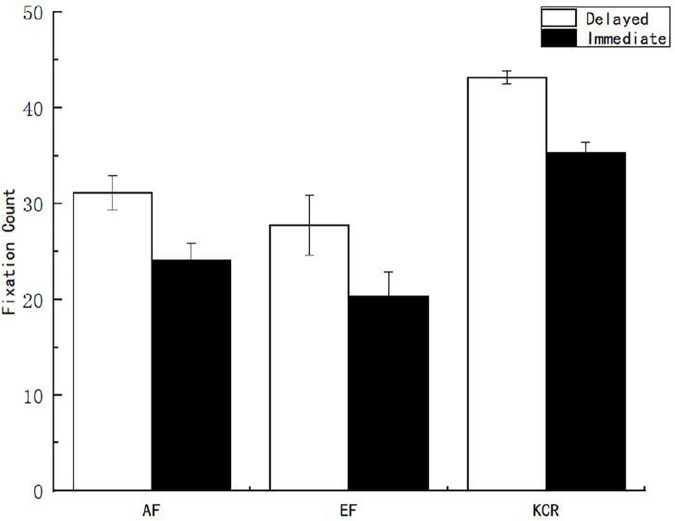
Fixation count of learners on the interest area of the stem under different feedback conditions.

**FIGURE 6:**
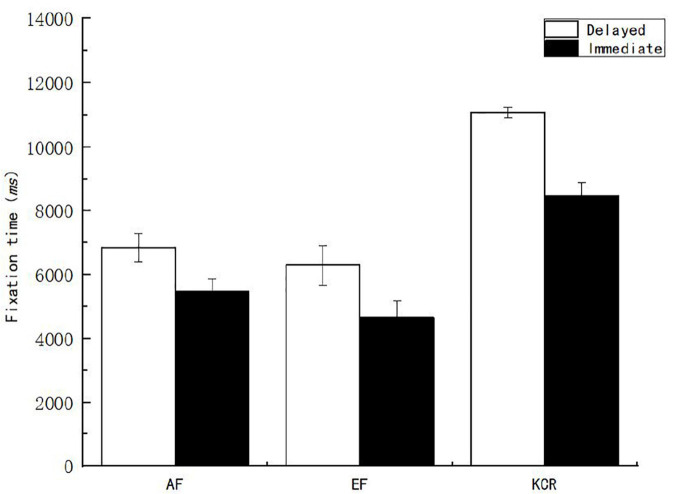
Fixation time of learners on the interest area of the stem under different feedback conditions.

Second, to explore the differences in fixation behaviors of subjects in the interest area of the stem under different feedback times and contents, variance analysis was performed on pupil size, fixation time, and fixation count. The results showed that the main effects of feedback time and content on pupil size were not significant (*p* > 0.05), while the main effects on fixation time and fixation count were significant. The main effect of feedback time on the fixation count in the stem interest area [*F*_(1, 151)_ = 18.183, *p* < 0.05, η^2^*_*p*_* = 0.107] showed that the fixation count in the delayed feedback group was significantly higher than that in the immediate feedback group. The main effect of feedback time on the fixation time of the stem interest area [*F*_(1, 151)_ = 18.698, *p* < 0.05, η^2^*_*p*_* = 0.110] showed that the fixation time of the subjects in the delayed feedback group was significantly higher than that in the immediate feedback group. The main effect of feedback content on the fixation count in the stem interest area was significant [*F*_(2, 151)_ = 27.434, *p* < 0.05, η^2^*_*p*_* = 0.267]. The main effect of feedback content on fixation time in the stem interest area [*F*_(2, 151)_ = 38.574, *p* < 0.05, η^2^*_*p*_* = 0.338] was also significant. After further multiple comparisons of the feedback content, it was found that the count and time of fixation in the stem area of the knowledge of the correct answer feedback group were significantly higher than those in the AF and EF groups (Figures 5, 6).

### Fixation Behavior of Learners on Feedback Interest Areas Under Different Feedback Conditions

First, the subjects performed a descriptive statistical analysis on the eye movement indexes of the feedback interest area, and the results are shown in Table 5.

**TABLE 5:** Descriptive statistical analysis.

	**IM-AF**		**IM-EF**		**IM-KCR**		**DE-AF**		**DE-EF**		**DE-KCR**	
**Dependent variable**	***M***	***SD***	***M***	***SD***	***M***	***SD***	***M***	***SD***	***M***	***SD***	***M***	***SD***
Pupil size	698.31	222.54	647.78	189.71	719.46	222.70	631.60	159.49	619.36	150.08	634.17	185.10
Fixation time (*ms*)	5562.82	2006.42	6675.24	2598.86	3118.25	2052.71	4486.94	2270.45	7291.75	3232.13	1683.08	861.49
Fixation count	27.46	8.94	34.15	13.23	11.63	5.78	21.90	9.22	38.30	16.42	7.64	3.36
Regression count	4.04	1.38	3.92	1.51	3.85	1.45	3.70	1.58	4.43	1.77	3.10	1.34

**FIGURE 7:**
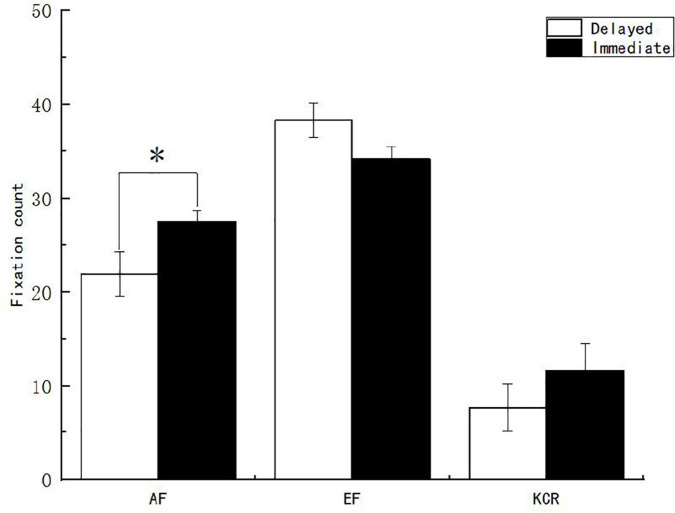
Fixation count of learners on the feedback interest area under different feedback conditions. *Significant correlation at the level of 0.05 (bilateral).

Second, to explore the differences in the fixation behavior of subjects in the feedback interest area with different feedback times and content, variance analysis was conducted on the pupil size, fixation time, fixation count, and regression count from the question stem interest area to the feedback interest area. The results showed that the main effect of feedback time on the fixation count in the feedback interest area was not significant (*p* > 0.05). The main effect of feedback content on the fixation count of the feedback interest area [*F*_(2, 151)_ = 84.665, *p* < 0.05, η^2^*_*p*_* = 0.529] was significant. After multiple comparisons of feedback content, it was found that the fixation count of the EF group was significantly higher than that of the AF group, while those of the AF group were significantly higher than those of the knowledge of the correct response feedback group (Figure 7). The interaction between feedback time and feedback content on the fixation count of the feedback interest area was significant [*F*_(2, 151)_ = 3.244, *p* < 0.05, η^2^*_*p*_* = 0.041]. Further simple effect analysis shows that under the condition of AF, the fixation count of the immediate feedback group was significantly larger than that of the delayed feedback group.

**FIGURE 8:**
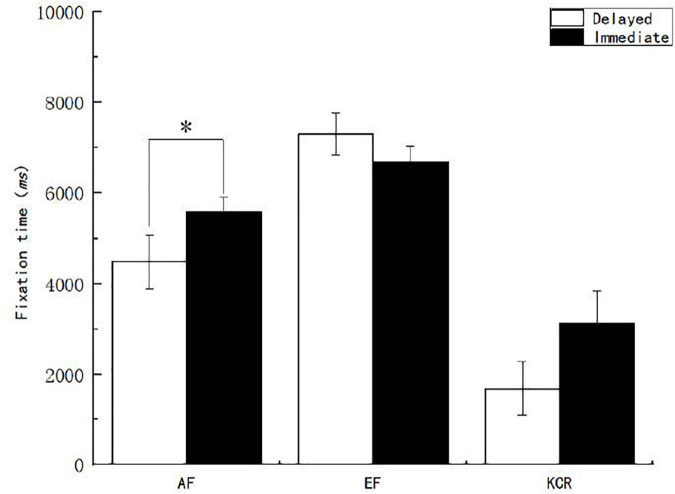
Learners’ fixation time on the feedback interest area under different feedback conditions. *Significant correlation at the level of 0.05 (bilateral).

The main effect of feedback time on the fixation time of the feedback interest area was not significant (*p* > 0.05). The main effect of feedback content on the fixation time of the feedback interest area [*F*_(2, 151)_ = 52.738, *p* < 0.05, η^2^*_*p*_* = 0.411] was significant. After further multiple comparisons of feedback content, it was found that the fixation time of the EF group was significantly higher than that of the AF group, while that of the AF group was significantly higher than that of the KCR feedback group (Figure 8). The interaction between feedback time and feedback content on the fixation time of the feedback interest area was significant [*F*_(2, 151)_ = 3.004, *p* < 0.05, η^2^*_*p*_* = 0.038]. Further simple effect analysis showed that under the condition of AF, the fixation time of the immediate feedback group was significantly longer than that of the delayed feedback group.

**FIGURE 9:**
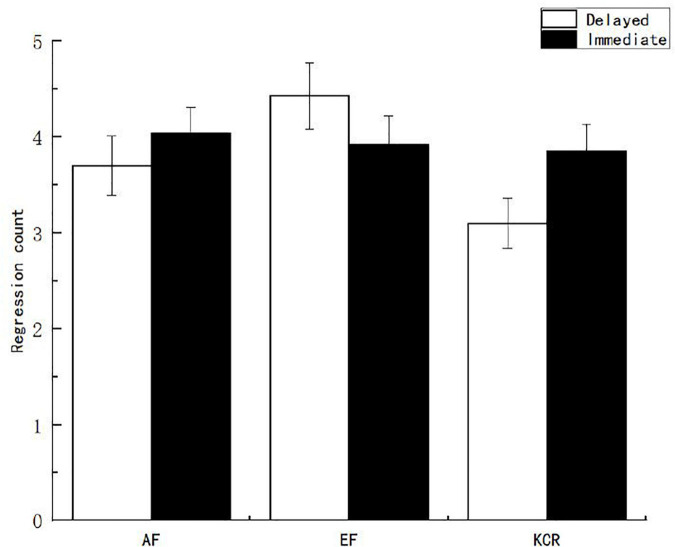
Regression count of learners on the feedback interest area under different feedback conditions.

The main effect of feedback time on the regression count in the feedback interest area was not significant (*p* > 0.05). However, the main effect of feedback content on the regression count in the feedback interest area [*F*_(2, 151)_ = 4.090, *p* < 0.05, η^2^*_*p*_* = 0.051] was significant. After multiple comparisons of feedback content, it was found that the regression count in the AF group and the EF group was significantly higher than that in the knowledge of the correct response feedback group (Figure 9).

## Discussion

### Influence of Feedback Time on Multimedia Academic Performance and Its Mechanism of College Students

Most previous studies on feedback timing have found that performance in the immediate feedback condition is higher than that in the delayed feedback condition (Miller, 2009; Van der Kleij et al., 2012), but some studies have found that learners achieve better transfer performance under delayed feedback conditions (Smith and Kimball, 2010). In this study, we not only found that the post-test scores of the immediate feedback group were significantly better than those of the delayed feedback group but also found that there were significant differences in the subjective reported germane cognitive load of the delayed feedback group and the fixation time and fixation count of the feedback interest area.

According to the cognitive load theory, when learners allocate their attention to learning-related content, their cognitive resources can be effectively utilized. Therefore, they have a higher germane cognitive load. When learners allocate their attention to content irrelevant to learning, they allocate limited resources to materials that are not helpful to learning, which will increase the external cognitive load. Therefore, it can be concluded from the results of this study that compared with delayed feedback, immediate feedback may be due to the increase in germane cognitive load (fixation time and count on the feedback interest area) and the decrease in external cognitive load (fixation time and count on the problem stem interest area), which is conducive to the improvement of academic performance.

The results of our study are consistent with the results of most previous studies, which not only accord with our initial research hypothesis but also support the hypothesis of cognitive load theory on instructional design.

### Influence of Feedback Content on Multimedia Academic Performance and Its Mechanism of College Students

Regarding the feedback content, some studies found that the performance would be better under the condition of EF (Van der Kleij et al., 2015), while some studies found that the more detailed the feedback content was, the greater the cognitive load was, and the more detrimental to learning (Van der Kleij et al., 2012; Zhang, 2015). In this study, we found that the differences in feedback content had significantly different effects on the post-test scores, motivation, related cognitive load of subjective reports, and fixation behavior in the question block interest and feedback interest areas of the subjects. In addition, our study also found that the related cognitive load of subjective reports of learners played a mediating role between AF and post-test performance. In other words, providing AF based on responses of the learners promoted effective cognitive processing of learners about learning materials, thus improving their performance. Although this study did not find a mediating role of motivation between feedback types and academic performance, this result partially supported our research hypothesis 4 and explained the mechanism of feedback, at least from the perspective of cognitive load.

In past research, multimedia learning feedback on whether to promote learning, what kind of feedback can improve learning, and who can promote more, these issues on the empirical research results are not consistent, and most previous studies use the scale measurement method, which is unable to perform real-time measurement of learners in the learning process after receiving feedback from the distribution of attention; this study used eye movement tracking technique with a combination of subjective reports of the participants and more objective measures of the process. According to the results of variance analysis, the feedback time and feedback content had significant main effects on the objective eye movement indices. Moreover, the η^2^ of six significant main effects varied from 0.051 to 0.529, with an average of 0.246, which was a large effect size. In contrast, feedback time and feedback content also had significant main effects on the post-test scores, motivation, and cognitive load, but from the perspective of η^2^, five significant main effects varied from 0.024 to 0.061, with an average of 0.0424, which was a smaller effect size. However, due to the limited conditions, all factors affecting the feedback effect were not included in this study. Therefore, the feedback effect mechanism can only be explained from the perspective of cognitive load. In future studies, the factors affecting the multimedia feedback effect and its mechanism should be further explored.

### Research Deficiencies and Prospects

This study has the following shortcomings, and we hope to further improve them in future research.

First, the selection of experimental materials and the particularity of the subjects limited the generalizability of the research results. The experimental material of this study is the quantitative rule part of graphic reasoning in the national civil service examination, which belongs to procedural knowledge. Therefore, to extend the conclusions of this study to other disciplinary knowledge or declarative knowledge, it is necessary to test whether the results of this study are consistent across disciplines.

The subjects in this study were all students from normal universities, mostly female students. Therefore, the generalization of the results of this study to other groups of subjects should be carefully considered. Future studies can be conducted with a wider group of subjects to verify and enrich the results of this study.

Second, this study only discusses the effects of feedback time and feedback content on multimedia learning from the perspective of educators but does not further explore the effects of individual characteristics and task characteristics of the learners on the feedback effect. Future research can start from the task, individual, and feedback characteristics and further explore what kind of feedback is best for which individual to complete what task, and why, to provide more empirical evidence for multimedia learning and teaching.

## Conclusion

The main conclusions of this study are as follows. In terms of feedback time, the academic performance of immediate feedback was significantly better than that of delayed feedback. The reason may be that, compared with delayed feedback, immediate feedback increases the germane cognitive load while reducing the external cognitive load.

In terms of feedback content, the AF proposed in this study was significantly better than the EF and KCR feedback in academic performance. Compared with EF and KCR feedback, AF improved academic performance by increasing germane cognitive load.

Compared with the KCR feedback, AF may also reduce external cognitive load and increase motivation.

## Data Availability Statement

The raw data supporting the conclusions of this article will be made available by the authors, without undue reservation.

## Ethics Statement

The studies involving human participants were reviewed and approved by the Ethical Committee for the Psychological Research of the Northeast Normal University. The patients/participants provided their written informed consent to participate in this study.

## Author Contributions

LH-D was the master’s advisor of ST and gave full guidance and participation in the writing and modification of the research article. The innovative idea of adaptive feedback in the manuscript also came from the guidance of LH-D. Both authors contributed to the article and approved the submitted version.

## Conflict of Interest

The authors declare that the research was conducted in the absence of any commercial or financial relationships that could be construed as a potential conflict of interest.

## Publisher’s Note

All claims expressed in this article are solely those of the authors and do not necessarily represent those of their affiliated organizations, or those of the publisher, the editors and the reviewers. Any product that may be evaluated in this article, or claim that may be made by its manufacturer, is not guaranteed or endorsed by the publisher.
